# The Puncture, Aspiration, Injection and Re-aspiration (PAIR) Technique of Hepatic Hydatid Cyst: A Case Report and Review of Literature

**DOI:** 10.7759/cureus.101231

**Published:** 2026-01-10

**Authors:** Abelardo Broceta, Ambika Kapil, Sahar S Abdelmoneim, Dariex Rodriguez, Carlos M Ramos Pachon

**Affiliations:** 1 Internal Medicine/Gastroenterology, Larkin Community Hospital, Miami, USA; 2 Osteopathic Medicine, Nova Southeastern University Dr. Kiran C. Patel College of Osteopathic Medicine, Davie, USA; 3 General Internal Medicine, Larkin Community Hospital, Miami, USA; 4 General Internal Medicine/Cardiovascular Medicine, Assiut University Hospital, Assiut, EGY; 5 Internal Medicine, Larkin Community Hospital, Miami, USA

**Keywords:** albendazole, echinococcus, extrahepatic, hepatic hydatid cyst, pair technique ruptured cyst

## Abstract

Hydatid disease, caused by *Echinococcus granulosus*, commonly affects the liver and can lead to complications such as rupture, secondary infection, and anaphylaxis. While surgery is the traditional treatment for complicated cases, minimally invasive techniques are emerging as viable alternatives. We report a case of a 27-year-old female with a history of gastritis and a pancreatic cyst who presented with acute diffuse abdominal pain and nausea. Imaging revealed an 11.8×8.8×5.2 cm cystic structure in the left hepatic lobe, consistent with a hydatid cyst, with free intraperitoneal fluid concerning for an impending or near rupture. The patient underwent percutaneous drainage of the cyst (puncture, aspiration, injection, and re-aspiration, PAIR technique), yielding 300 cc of serosanguinous fluid. She was started on albendazole therapy and closely monitored. Over nine days of hospitalization, her condition improved, drainage output decreased, and she was discharged in stable condition with a structured follow-up plan. Our report demonstrates that percutaneous drainage with adjunctive albendazole therapy is a feasible and effective alternative to surgery in hemodynamically stable patients with ruptured hepatic hydatid cysts. Further studies are warranted to refine patient selection criteria and optimize long-term outcomes.

## Introduction

Hydatid disease, caused by the parasitic tapeworm *Echinococcus granulosus*, is a zoonotic infection that most commonly affects the liver and lungs, though it can occur in nearly any organ. Humans become accidental intermediate hosts through ingestion of eggs in contaminated food, water, or soil, especially in regions where livestock and dogs live in close proximity to humans. The disease is endemic in rural areas of South America, Eastern Europe, Africa, the Middle East, and Central Asia, but sporadic cases are reported worldwide due to increased travel and migration [1,2].

Globally, an estimated 2 to 3 million people are affected, though this likely underestimates the true burden because comprehensive epidemiological data are not available for all endemic regions. In highly endemic areas, incidence can reach up to 50 cases per 100,000 person-years, with prevalence approaching 10%. Factors such as low socioeconomic conditions, poor hygiene, and unsafe livestock slaughtering practices contribute to higher transmission rates [3-5].

Hepatic hydatid cysts represent the majority of cases and are often asymptomatic, discovered incidentally on imaging. Complications such as cyst rupture, secondary infection, biliary obstruction, or anaphylactic reactions are uncommon but can present as acute, potentially life-threatening events [6]. Diagnosis relies on imaging modalities, including ultrasound (US) and computed tomography (CT), which provide critical information for treatment planning and monitoring response. While surgery has traditionally been the standard therapy for hepatic hydatid cysts, minimally invasive techniques such as puncture, aspiration, injection, and re-aspiration (PAIR), combined with albendazole therapy, have emerged as effective alternatives in selected patients, offering high technical success rates and lower morbidity [7-9].

This report describes a 27-year-old female presenting with a large hepatic hydatid cyst and acute abdominal pain. It highlights the importance of a multidisciplinary approach in complicated cases and demonstrates the successful application of minimally invasive therapy in a high-risk scenario, adding to the growing literature supporting PAIR as a treatment option. We report this case to highlight the successful use of the PAIR technique in managing a large, near-ruptured hepatic hydatid cyst, demonstrating the feasibility of minimally invasive treatment in select high-risk scenarios.

## Case presentation

A 27-year-old female with a past medical history of gastritis and a pancreatic cyst, first diagnosed on MRI three months prior, presented to the emergency department with a two-day history of abdominal pain, associated with nausea after eating. The patient characterized the pain as diffuse and cramp-like, rating its intensity as 10 out of 10 on the pain scale (with 10 being the most severe). The patient's vital signs were within normal range as the temperature was 36.9°C (oral), heart rate was 80 beats per minute, respiratory rate was 16 breaths per minute, blood pressure was 126/90 mmHg, and oxygen saturation was 99% on room air.

**Figure 1:**
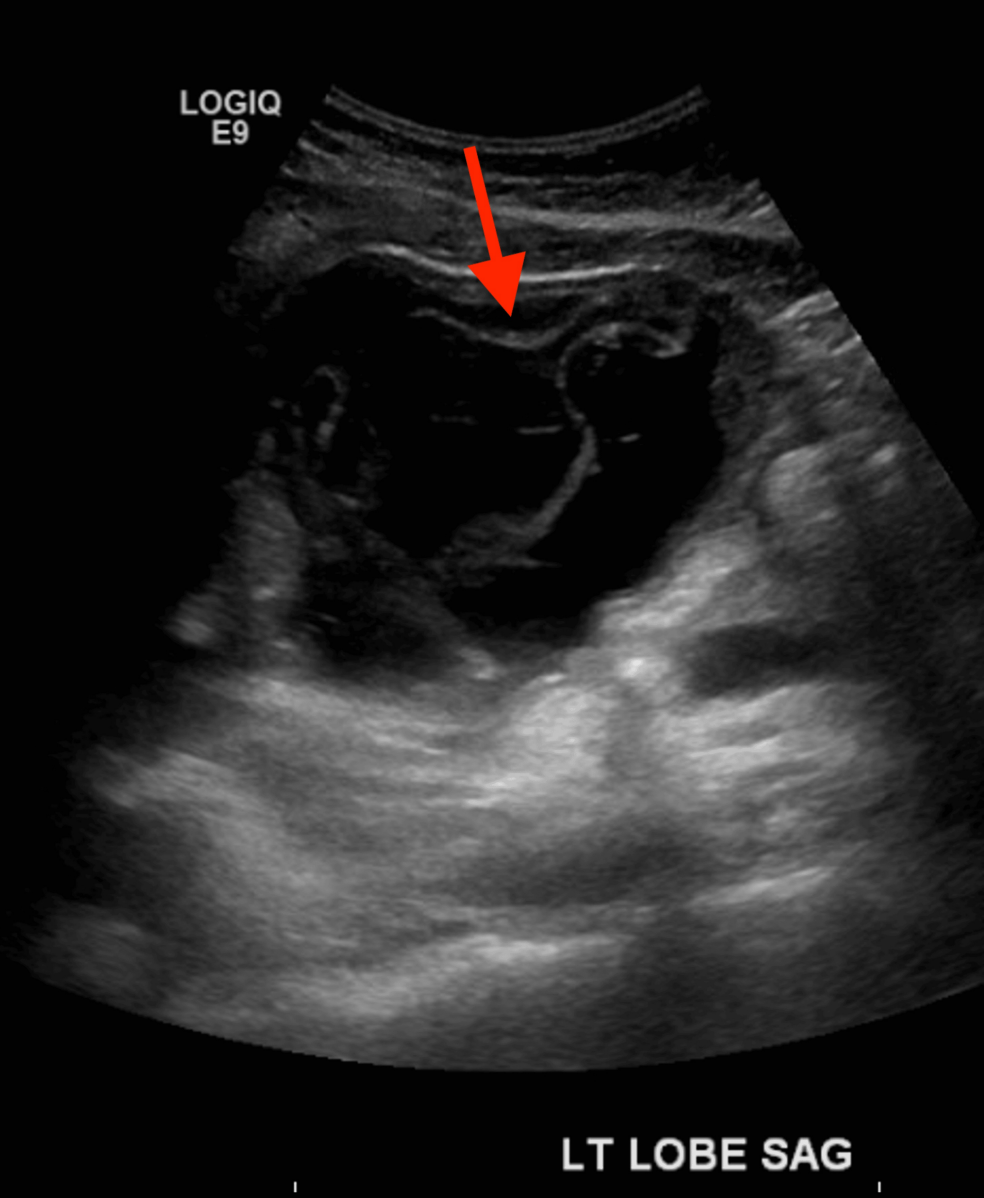
Abdominal ultrasound Abdominal ultrasound demonstrating a complex cystic structure in the left hepatic lobe measuring 11.6×8×8 cm (red arrow), consistent with a hydatid cyst. The size and morphology, along with the patient's clinical history, raised concern for potential rupture, guiding the decision for PAIR intervention. PAIR - puncture, aspiration, injection, and re-aspiration

Physical examination revealed a soft abdomen with mild diffuse tenderness, most pronounced in the epigastric region, with no signs of guarding or rebound tenderness. There was no evidence of jaundice, and the rest of her physical exam was unremarkable. Additionally, the patient noted a recent history of traveling to South America and having close contact with livestock and dogs. Given her recent travel to South America and close contact with livestock and dogs, it is likely that the patient contracted *Echinococcus granulosus* through inadvertent ingestion of eggs from contaminated sources. Otherwise, the patient denied any recent proximity to sick contacts. Initial abdominal US demonstrated a complex cystic structure in the left hepatic lobe measuring 11.6x8x8 cm, as demonstrated in Figure 1. Subsequent CT scan of the abdomen and pelvis showed an 11.8x8.8x5.2 cm irregular hypodense cystic structure in the left hepatic lobe, which resembled a hydatid cyst. Additionally, it was noted that there was free fluid around the gallbladder fossa and in the right paracolic gutter, extending into the pelvis. These findings raised concerns about a ruptured cyst. As a result, the patient was admitted to the hospital for further management. The discovery of liver cysts on the CT scan prompted further laboratory evaluation, which revealed hypereosinophilia, neutrophilia, and a positive *Echinococcus *IgG result (Table 1). The presence of hypereosinophilia is consistent with parasitic infection and further supports the suspicion of hydatid disease.

**Table 1:** Hospital laboratory values This table summarizes the patient’s daily complete blood count (CBC), pertinent comprehensive metabolic panel (CMP), and differential values from day 0 to day 12. Notably, the labs demonstrate an initial hemoconcentration with subsequent normalization of hemoglobin and hematocrit, stable red cell indices, a mild late leukocytosis with transient neutrophilia, persistent eosinophilia through day 10, and a gradual rise in platelet count consistent with reactive thrombocytosis. Liver enzymes and lipase levels remained within normal limits. WBC - white blood cell; RBC - red blood cell; MCV - mean corpuscular volume; MCH- mean corpuscular hemoglobin; MCHC- mean corpuscular hemoglobin concentration; MPV - mean platelet volume; AST - aspartate aminotransferase; ALT - alanine aminotransferase; ALP - alkaline phosphatase; ↑ - above reference range; ↓ - below reference range; N/A - not available

Laboratory tests	Day 0	Day 3	Day 5	Day 6	Day 7	Day 8	Day 10	Day 11	Day 12	Reference range
Hemoglobin (g/dL)	17.1 ↑	11.5 ↓	12.5	13.9	12.9	12.8	12.9	12.8	12.4	12.0–16.0
Hematocrit (%)	50.9 ↑	34.7 ↓	37.7	42.7	39.5	39.2	38.9	38.6	37.2	36–46
WBC (x10⁹/L)	9.9	7.2	7.2	7.8	8.3	8.6	9.4	9.5	13.6 ↑	4.5–11.0
MCV (fL)	91.9	94.0	93.1	94.3	92.7	95.1	92.4	91.0	91.6	80–100
MCH (pg/cell)	30.9	31.2	30.9	30.7	30.3	31.1	30.6	30.2	30.5	27–31
MCHC (g/dL)	33.6	33.1	33.2	32.6	32.7	32.7	33.2	33.2	33.3	32–36
Platelets (×10³/μL)	294	223	295	355	355	358	369	396	365	150–450
MPV (fL)	11.0	10.9	10.4	9.9	9.8	10.1	9.5	10	10	7–12
Neutrophil (%)	90.3 ↑	67.9	59.0	60.8	56.2	50.5	53.3	77.1 ↑	N/A	30–75
Lymphocyte (%)	4.6 ↓	15.0	20.1	21.0	24.2	26.0	19.6	12.4 ↓	28	20–45
Eosinophil (%)	N/A	7.6 ↑	9.7 ↑	10.0 ↑	9.3 ↑	11.7 ↑	18.7 ↑	5.9	N/A	0–6
Glucose (mg/dL)	106	104	91	92	127 ↑	N/A	N/A	92	92	70–100
Lipase (U/L)	88	N/A	N/A	N/A	N/A	N/A	N/A	N/A	N/A	0–160
AST (U/L)	35 ↑	42 ↑	27	28	31	N/A	N/A	28	31	10–34
ALT (U/L)	28	50 ↑	42	39	39	N/A	N/A	41	41	10–130
ALP (U/L)	90	85	79	72	76	N/A	N/A	87	70	35–104

A multidisciplinary team of gastroenterologist, primary care, infectious disease specialist, interventional radiologist (IR), and pain management specialist were involved in the patient's coordinated care. Additionally, the patient was started on a regimen of inpatient medications which included enoxaparin 40 mg subcutaneously once daily, pantoprazole 40 mg IV push, butalbital/acetaminophen/caffeine (50 mg/325 mg/40 mg) as needed every six hours, cyclobenzaprine 10 mg daily as needed, acetaminophen 650 mg every six hours as needed, and melatonin 5 mg at bedtime as needed. Albendazole 400 mg twice daily for about four days was initiated per infectious disease recommendations before percutaneous aspiration of the cyst by the IR team utilizing the PAIR technique. Peri-procedural antibacterial prophylaxis was not explicitly documented in the medical record; however, the patient experienced no post-procedural infectious complications, and all cyst fluid cultures remained negative.

**Figure 2:**
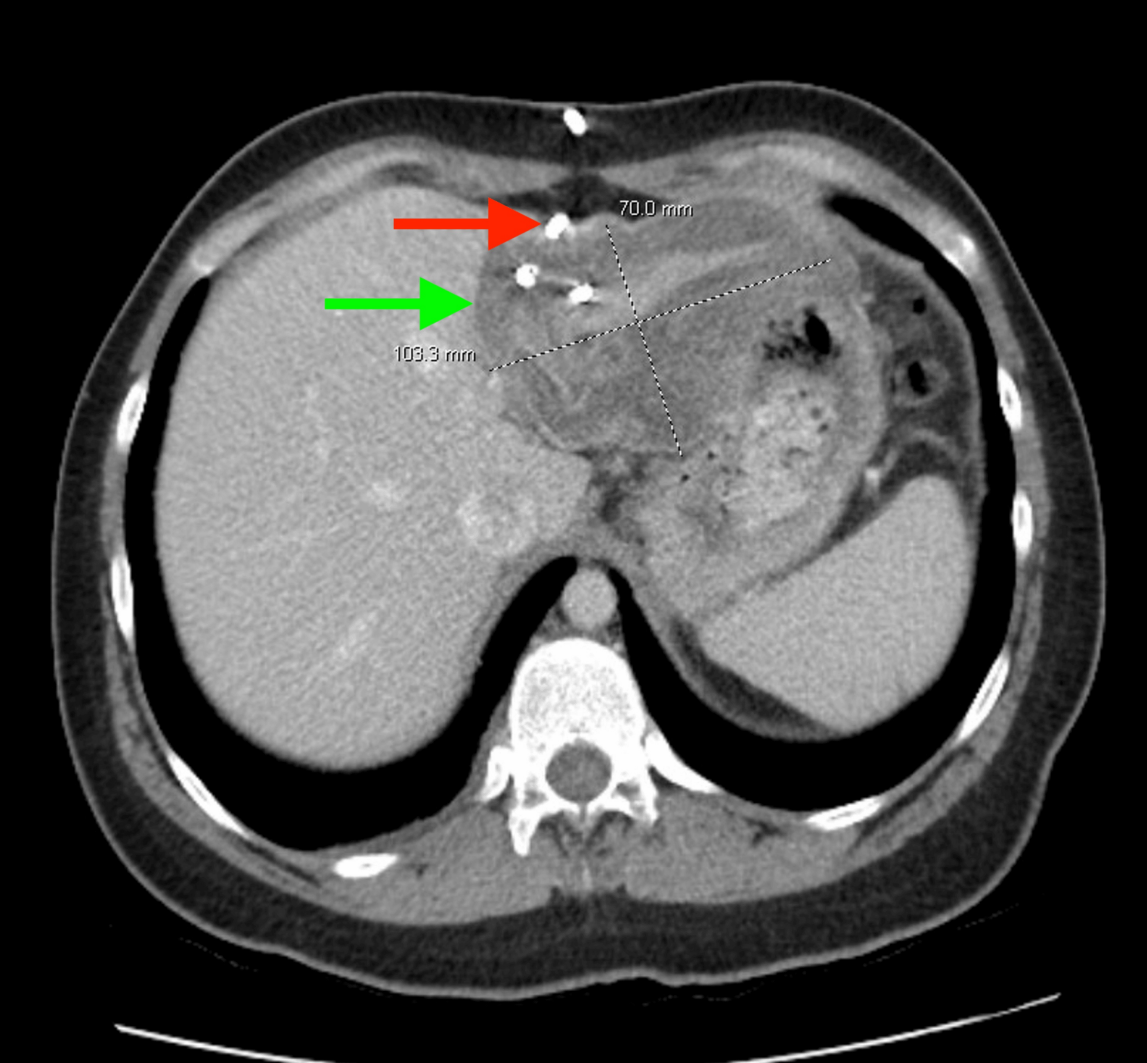
CT abdomen and pelvis status after percutaneous drainage of hepatic cyst CT abdomen and pelvis status post percutaneous drainage of hepatic cyst, showing an interval decrease in size of the collection in the left lobe of liver (green arrow), with a pigtail drainage catheter (red arrow) seen within the collection. The collection had decreased from the prior measurement of 11.8x8.8 cm to a 10x7 cm.

The patient underwent US-guided drainage catheter placement using the PAIR technique (Flexima™ APD Loop 10 Fr) for the complex cyst in the left hepatic lobe. Approximately 300 cc of serosanguineous fluid was aspirated without complication. Following aspiration, a scolicidal agent (hypertonic saline) was instilled into the cyst cavity and subsequently re-aspirated, in accordance with the PAIR protocol. The cyst fluid was sent for routine bacterial, fungal, and mycobacterial cultures, all of which were negative. No cytologic or histopathologic analysis of the aspirated fluid was performed, as the specimen was submitted only for routine cultures (Table 2). A follow-up CT scan of the abdomen and pelvis (Figure 2) demonstrated a slight decrease in the size of the collection, from 11.2×10 cm to 10×7 cm, and reduced fluid volume, consistent with gradual interval improvement. The patient's pain continued to improve, and albendazole was maintained post-procedure to complete the recommended treatment course of 3-6 months.

**Table 2:** Aspirated cyst fluid analysis (300 cc of serosanguineous fluid) This table summarizes the gross appearance and cellular profile of the aspirated hepatic cyst fluid, including fluid color, red and white blood cell counts, differential distribution, macrophage presence, and *Echinococcus* IgG results. The fluid demonstrates elevated WBCs and neutrophils consistent with an inflammatory response, markedly elevated RBCs reflecting hemorrhagic content, and positive *Echinococcus* IgG supporting the diagnosis of hydatid cyst. ↑ = above reference range

Laboratory marker	Patient value	Reference/normal
*Echinococcus* IgG	Positive	Negative
Fluid Appearance	Bloody	Clear/straw-colored
Fluid WBC (/µL)	195 ↑	<50–100
Fluid RBC (/µL)	87,000 ↑	<5
Fluid neutrophil (%)	93 ↑	<25
Fluid lymphocyte (%)	7 ↑	None
Fluid macrophage	Present	None

The patient's condition showed great improvement, and drainage output decreased to less than 30 cc per day by the seventh day of admission. The surgical and infectious disease teams recommended continuing the drainage, flushing the catheter with 10 cc of sterile saline 12 times daily. On the eighth day, the drainage catheter was removed after confirmation of adequate drainage, and the catheter site was cleaned with sterile swabs and gauze, and a Tegaderm dressing was applied.

Follow-up showed the dressing site to be clean, dry, and intact, with no hematoma or discharge. The patient was discharged in a stable condition on the ninth day. She was instructed to continue albendazole treatment for the hydatid cyst, with follow-up appointments scheduled for one week and one month post-discharge with her primary care provider for ongoing monitoring, as well as with the infectious disease team for ongoing management of the hydatid cyst. The patient was also instructed to visit the pharmacy monthly for medication refills and to report any concerning symptoms, such as fever or worsening pain, to her healthcare providers. The outpatient clinic follow-up was uneventful. 

## Discussion

Hydatid disease, caused by the larval stage of *Echinococcus granulosus*, primarily affects the liver and lungs and can manifest with a variety of clinical presentations. Humans acquire the infection by ingesting eggs from contaminated food or water, leading to cyst formation in affected organs [8]. While hepatic involvement is most common, rupture of a hydatid cyst, such as in this case, represents a serious complication with potentially life-threatening consequences.

Ultrasound remains the gold standard for imaging abdominal cystic echinococcosis, but CT of the abdomen and pelvis has demonstrated 94% accuracy in distinguishing hydatid cysts from pyogenic or amebic liver cysts [10]. The PAIR technique involves puncturing the cyst, aspirating its contents, injecting a scolicidal agent (in this case, hypertonic saline) to inactivate the parasite, and then re-aspirating the fluid [9]. This method effectively reduces viable parasites and minimizes the risk of dissemination.

In most published cases, hepatic hydatid cysts present as incidental findings or with nonspecific symptoms such as mild abdominal discomfort. For instance, Sahin et al. described a patient with a giant hepatic hydatid cyst that remained asymptomatic until mass effect on adjacent structures necessitated surgical removal [11]. In contrast, our patient presented with a large, near-ruptured cyst. Unlike the surgical approaches described in prior cases, this patient was successfully managed using a minimally invasive technique, US-guided drainage utilizing the PAIR technique, combined with albendazole therapy. This highlights a potentially less morbid alternative to surgery in select patients and emphasizes the role of interventional radiology in managing complicated hydatid cysts. Successful percutaneous treatment was determined by significant reductions in cyst size, disappearance of the fluid component, and eventual solidification of the cyst with no further growth on follow-up imaging.

A key aspect of this case involves the initial imaging findings. Figure 1 illustrates the abdominal US measurements of the cyst, recorded as 11.6×8×8 cm, whereas a subsequent non-contrast CT scan of the abdomen measured the cyst at 11.8×8.8×5.2 cm. This discrepancy can be attributed to several factors. Ultrasound is operator-dependent, and variations in measurements can result from probe angle and imaging plane. The anteroposterior (AP) diameter may appear larger due to compression from adjacent structures during real-time imaging, as the probe applies pressure [10]. Additionally, fluid-filled cysts often exhibit posterior acoustic enhancement, which can artificially increase the perceived size. In contrast, CT imaging provides more consistent measurements. The difference in the smallest dimension (8 cm on US versus 5.2 cm on CT) suggests that the US may have included adjacent tissue or overstated the cyst wall. Follow-up imaging demonstrated a positive treatment response. Figure 2 displays a CT scan of the abdomen and pelvis, revealing a decrease in cystic collection size from 11.2x10 cm to 10x7 cm, alongside a reduction in fluid volume and demonstrating progressive resolution of the cyst post-drainage. Notably, the initial CT abdomen measurement of 11.8x8.8x5.2 cm serves as a reference for size progression, further corroborating the therapeutic efficacy of PAIR in this case.

Significant reductions in the cyst's size, disappearance of fluid component volume, and eventual solidification of the cyst, with no growth in size during follow-up, were the main criteria for successful percutaneous hydatid cyst treatment. A summary of selected studies in the literature emphasizing the PAIR technique for the management of hydatid cyst is listed in Table 3 [12-16]. The studies in this review, selected from the past five years, focus on the PAIR technique for managing hydatid cysts, highlighting its efficacy, safety, complication rates, and comparative effectiveness against surgical or medical management. Our case is notable for the successful management of a large, near-ruptured hepatic hydatid cyst, which is less commonly reported in the literature. PAIR has demonstrated advantages such as shorter hospital stays, lower major complication rates, and technical success in treating cysts, making it a promising alternative. However, surgery remains the gold standard for treatment, particularly in cases of large, multiple, or complicated cysts, such as those with cysto-biliary fistulas, rupture, or extensive organ involvement. Surgery is preferred when complete cyst removal is necessary to minimize recurrence risk and prevent complications that PAIR may not adequately address [9].

**Table 3:** Selected studies from the past five years emphasizing the PAIR technique for hydatid cyst management, with case-specific highlights Cyst diameters are reported as the largest dimension measured. Complication rates reflect study-reported major adverse events (sepsis, hemorrhage, cyst rupture, or incomplete removal). PAIR - puncture, aspiration, injection, re-aspiration

Study	Sample size	Age group	Techniques	Cyst diameter	Outcomes	Case highlights
Özdemir M, et al. 2023 [12]	58 cysts	18-80 years (mean 40±17.7)	Percutaneous treatment	>10 cm	Technique is efficacious and has low complication rates	Most cases were uncomplicated, not near-ruptured cysts
Erkmen F, et al. 2024 [13]	209 patients	18-77 years	Surgery in 87.5% vs. PAIR in 12.5%	≥9.5 cm	Biliary fistula most prevalent complication; PAIR complications 28.4% vs surgery 65.4%	Few near-ruptured cysts treated with PAIR
Akhan O, et al. 2020 [14]	40 patients	Not specified	PAIR vs catheterization	Not specified	Technical success 91.9%; lower major complication rates; shorter hospital stays; cysto-biliary fistula and recurrence 2.94% in PAIR	Most cases were elective or uncomplicated cysts
Elmoghazy W, et al. 2023 [15]	57 patients (98 cysts)	Adults (18-72 yrs)	Open surgery vs. minimally invasive (laparoscopic/robotic)	4-23 cm	No mortality; no recurrence; overall complication rate 33.3% (mostly minor); bile leak 14%; the minimally invasive group had a significantly shorter hospital stay	Demonstrated safety and feasibility of minimally invasive surgery for a broad range of cyst sizes and locations; provided a contemporary comparison of surgical approaches
Sokouti M, et al. 2019 [16]	57 studies (6 prospective, 51 retrospective)	5-87 years	23 PAIR vs 34 laparoscopic surgical	Variable across studies	Higher cure rate with PAIR; low complication/mortality; higher recurrence noted	Most cysts were near-ruptured but not acutely life-threatening; no cases required urgent intervention as in our high-risk presentation.

Albendazole was chosen for this patient because it remains the first-line benzimidazole antiparasitic for hepatic cystic echinococcosis [17,18]. It is metabolized in the liver to albendazole-sulfoxide, the active form, which disrupts the parasite's glucose uptake and energy metabolism, causing death of protoscoleces and degeneration of the cyst germinal layer [19]. Continuous oral dosing of 10-15 mg/kg/day (up to 400 mg twice daily) for 3-6 months is recommended for uncomplicated or percutaneously treated cysts [17,19]. Complete cyst disappearance is uncommon, but sterilization, solidification, or significant size reduction is often achieved [17,19]. Peripheral eosinophilia may gradually normalize, but its resolution should be interpreted alongside imaging and serology, as parasitic antigens and cyst wall calcification can persist despite clinical stability [18,19].

In the patient reported in this case, peripheral eosinophilia gradually normalized alongside reductions in cyst size and fluid volume, suggesting a direct correlation between laboratory trends and treatment efficacy. The patient's PAIR procedure yielded approximately 300 cc of aspirated fluid over the initial drainage period, with catheter output decreasing to less than 30 cc/day by the seventh day. She was discharged on the ninth day, consistent with shorter hospital stays reported in the literature (Table 3), further supporting the effectiveness of minimally invasive management combined with albendazole therapy. These findings align with previously reported outcomes of PAIR, demonstrating rapid clinical improvement, reduced procedural morbidity, and efficient cyst sterilization in selected patients.

Although PAIR shows clear therapeutic benefits, this case report has limitations. As a single-case study, the findings may not be generalizable to larger populations. Long-term follow-up is needed to assess recurrence rates and the durability of treatment effects. Variability in cyst features and patient selection can influence success, making direct comparisons with surgical intervention challenging. Larger studies with standardized protocols and extended follow-up are required to determine the optimal management approach for hydatid cysts.

## Conclusions

This case illustrates that PAIR combined with antiparasitic therapy may be a feasible and safe option for select patients with complicated hepatic hydatid cysts, even in the setting of suspected cyst instability, when careful patient selection and multidisciplinary coordination are employed. Although the presence of free intraperitoneal fluid raised concern for impending rupture, minimally invasive management in this patient resulted in favorable clinical and radiographic outcomes without complication. This underscores the importance of individualized risk assessment rather than reliance on cyst size or imaging findings alone when determining management strategies.

Future studies are needed to better define standardized criteria for PAIR eligibility in complicated hepatic hydatid cysts, including those with radiologic features suggestive of leakage or near rupture. Long-term follow-up and larger prospective cohorts would further clarify recurrence risk, optimal duration of antiparasitic therapy, and the role of adjunctive scolicidal agents in improving outcomes. Reporting such cases will continue to refine evidence-based, less invasive management strategies, while recognizing that surgery remains the standard definitive treatment for complicated cysts.
